# UV-Cured Inkjet-Printed Silver Gate Electrode with Low Electrical Resistivity

**DOI:** 10.1186/s11671-017-2300-9

**Published:** 2017-09-25

**Authors:** Honglong Ning, Yicong Zhou, Zhiqiang Fang, Rihui Yao, Ruiqiang Tao, Jianqiu Chen, Wei Cai, Zhennan Zhu, Caigui Yang, Jinglin Wei, Lei Wang, Junbiao Peng

**Affiliations:** Institute of Polymer Optoelectronic Materials and Devices, State Key Laboratory of Luminescent Materials and Devices, South China University of Technology, Guangzhou, 510640 People’s Republic of China

**Keywords:** Inkjet printing, UV curing, Silver gate electrode, Electrical resistivity, Low temperature

## Abstract

Inkjet-printed silver gate electrode with low electrical resistivity was fabricated by UV curing method. By adjusting the UV curing time and the distance between the samples and UV lamp, the effects of UV curing conditions on the electrical resistivity of the silver films were studied, and the lowest electrical resistivity of 6.69 × 10^−8^ Ω·m was obtained. Besides, the UV-cured silver films have good adhesion to the glass substrates, with adhesion strength of 4B (ASTM international standard). Our work offered an easy and low temperature approach to fabricate inkjet-printed silver electrodes with low electrical resistivity.

## Background

With the development of printed electronics, inkjet printing has attracted increasing attention from academic and industrial communities. Many works regarding the applications of inkjet printing on thin film transistors are conducted [1, 2]. Inkjet printing can not only reduce the process steps and material waste by drop-on-demand technique [3, 4], but also enable the direct patterning of devices [5]. Besides, low-temperature manufacture is becoming more and more important for the fabrication of electronic products. UV curing method is known as a low-temperature and fast curing method which can meet the demand for low-temperature manufacture of electronics.

Most previous works on printing electronics have focused on heat curing method [6–11]. However, heat curing method is normally performed at above 200 °C for more than 30 min in an effort to remove the organic residuals in the ink, which is undesirable for ever-growing flexible electronics that require low-temperature or even room-temperature manufacturing techniques. In addition, laser sintering [12], electrical sintering [13], and other methods [14, 15] are used to cure the inkjet-printed silver films in some works.

In this paper, silver nanoparticle ink was used to fabricate gate electrodes due to its good conductivity and chemical stability compared to copper. More importantly, the melting temperature of silver nanometer particles is much lower than that of bulk silver, which enables the low-temperature production of conductive films [14, 16]. Since the electrical resistivity of inkjet-printed silver gate electrodes is greatly affected by post-treatment process, the effects of UV curing conditions on the electrical resistivity of the silver films were investigated. Besides, the adhesion of UV-cured silver films was also measured by tape test. Finally, we discussed the differences between UV-cured films with heat-treated films.

## Methods

Glass was used as the substrate materials. To remove the surface contamination, these substrates were ultrasonicated in isopropyl alcohol, tetrahydrofuran, deionized water and isopropyl alcohol in sequence. The silver nanoparticle ink used in inkjet printing was DGP-40LT-15C purchased from Advanced Nano Products Co. Ltd. A Dimatix (DMP-2800) printer with a 10pL cartridge was used to print the desired films. During the printing, the substrate temperature of the printer was set at 30 °C, and the silver nanoparticle ink was printed onto the substrates with drop spacing of 35 μm. After the printing, the films were cured by a UV light curing system (IntelliRay UV0832, Uvitron International Inc.). The power of UV lamp in the system is 600 W.

*D* was defined as the distance between the silver films and the UV lamp during UV curing. When *D* = 37 cm, the films were cured at different UV curing times to study the effects of the UV curing time on the electrical resistivity: 180, 240, 360, and 480 s. To study the effects of D on the electrical resistivity, the films were cured at different distances when curing time was set to 180 s: 37, 29, 27, 25, and 23 cm. And then, we cured the silver film at different UV curing conditions to find out the optimum conditions based on the results above. Besides, the films were also heat-treated in air at different temperatures for comparison: 25, 70, 100, 120, and 140 °C.

The electrical resistivity of the films was calculated from *ρ* = R_s_ × h(*ρ*: electrical resistivity, *R*_s_: sheet resistance, h: the thickness of the films). The sheet resistance was measured by a digital four-probe tester (KDY-1, Guangzhou Kunde Co.Ltd). The thickness was measured by a step profiler (Dektak). A scanning electron microscopy (SEM, NOVA NANOSEM 430) with an energy dispersive X-ray spectrometer (EDS) was used to obtain the surface information and element content of the cured silver films. The 3D morphology images were characterized by an optical profiler (Veeco NT 9300).

## Experimental Principle

**Fig. 1:**
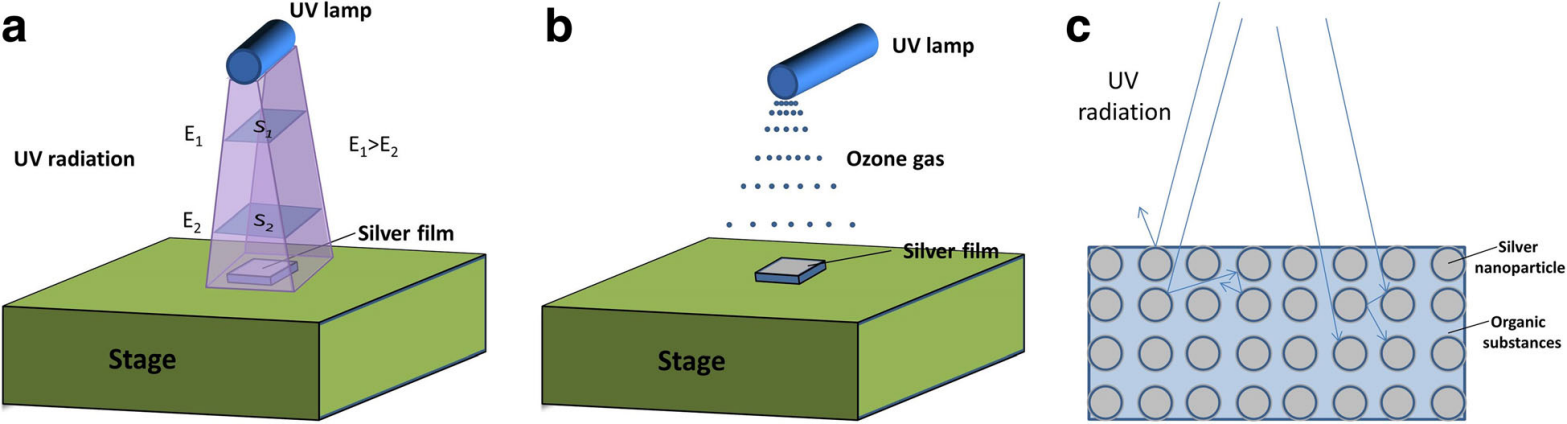
The schematic diagrams of UV curing method: (**a**) the intensity of UV irradiation at different distances; (**b**) the distribution of the ozone gas generated by UV irradiation; (**c**) the propagation of UV irradiation when silver film is being cured

Because oxygen in air will absorb UV radiation and be transformed into ozone gas which renders the rapid ultraviolet radiation attenuation in air [17, 18], the energy of UV radiation to which the silver films are exposed will decrease as the increase of *D* (*E*_1_ > *E*_2_). As shown in Fig. 1a, the intensity of UV radiation decreases when D increases ($\frac{E_1}{S_1}>\frac{E_2}{S_2}$). The concentration of ozone gas also decreases with the increasing D shown in Fig. 1b. Besides, ozone gas will react with silver films and generate silver oxide which will increase the electrical resistivity of the films.

Fig. 1c shows the mechanism of UV curing. When UV radiation reaches the surface of the untreated silver films, only a small part of the radiation penetrates into the film which may be trapped at a specific depth of the films, or get out of the film due to the reflection, or penetrate into deeper layers. The deeper the penetration depth is, the weaker UV radiation becomes. During this process, the radiation will be absorbed by the silver nanoparticles and organic substances, and then converts into heat [19, 20]. When heat gradually accumulates within the films, temperature will increase that leads to the removal of the organic substances. Moreover, the curing depth will be deeper and the removal of the organic substances will be promoted when D decreases, which means radiation becomes stronger.

## Results and Discussion

**Fig. 2:**
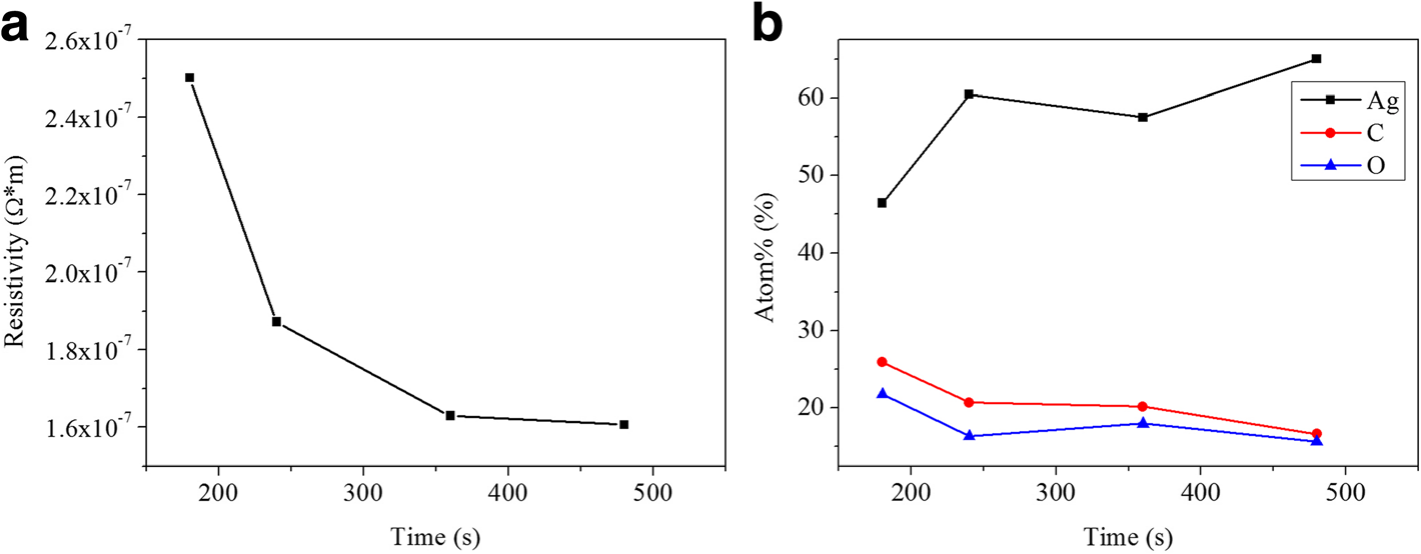
When *D* = 37 cm (**a**)resistivity versus UV curing time; (**b**) atomic relative content of elements of the films versus UV curing time

Figure 2a shows the influence of UV curing time on the electrical resistivity of the silver films when *D* = 37 cm. The electrical resistivity decreased dramatically when UV curing time increased up to 360 s. As the time continues to increase, it decreased slightly. Figure 2b illustrates the changes of atomic relative contents of the silver films as the increase of UV curing time when *D* = 37 cm. The atomic relative content of carbon and oxygen gradually decreased while that of silver increased, which meant the organic substances with high electrical resistivity were gradually removed. During this process, the curing degree increased and the electrical resistivity of silver films became lower. When UV curing time increased from 360 to 480 s, the slight decrease of electrical resistivity indicated that the curing degree at *D* = 37 cm was almost close to the maximum. Obviously, the UV radiation at *D* = 37 cm was not strong enough to remove more residual organic substances when UV curing time was over 360 s.

**Fig. 3:**
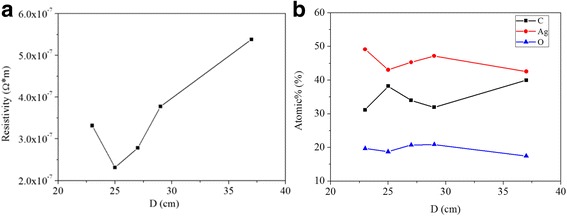
When UV curing time was 180 s: (**a**) resistivity versus distance; (**b**) atomic relative content of elements of the films versus distance

Figure 3a shows the effects of D on the electrical resistivity of the silver films when UV curing time is 180 s. When D decreased from 37 to 25 cm, the electrical resistivity decreased rapidly. Subsequently, the electrical resistivity increased when D decreased from 25 to 23 cm. The organic substances in the silver films were gradually removed when D decreased from 37 to 25 cm, contributing to the reduction of electrical resistivity.

Figure 3b shows the changes of the relative contents of silver, carbon and oxygen as a function of D when UV curing time is 180 s. As shown in Fig. 3b, when D decreased from 37 to 29 cm, the relative content of carbon decreased while that of oxygen slightly increased. While *D* decreased, the silver films were exposed to higher levels of UV radiation, which meant the curing depth became deeper, the UV curing and the generation of heat became faster. As a result, more organic substances were removed. So it made sense that the relative content of carbon decreased when *D* decreased from 37 to 29 cm.

When *D* decreased from 29 to 25 cm, the relative content of carbon increased while the relative content of oxygen slightly decreased. It indicated that the organic substances may be carbonized leading to the formation of the conductive carbon. When D decreased, the stronger UV irradiation induced a higher temperature of silver film. When *D* = 27 cm, the temperature was high enough to form conductive carbon because of the carbonization of organic substances. The carbon bridged the silver nanoparticles, giving rise to the decrease of electrical resistivity [13]. When D decreased from 27 to 25 cm, more carbon was formed between neighboring silver nanoparticles that caused the further reduction of electrical resistivity.

The relative content of carbon decreased rapidly while oxygen content increased when D decreased from 25 to 23 cm. Meanwhile, the electrical resistivity of the silver film decreased. There were two possible reasons for this phenomenon. The first one was the oxidation of silver nanoparticles. Sung Joon Kim et al. proposed that the amorphous silver oxide was formed on the silver film due to the reaction of ozone gas to the silver films [21]. The increasing relative content of oxygen when *D* = 23 cm indicates the oxidation of the silver film. When *D* decreased, the intensity of radiation became larger, and ozone gas was more possible to be generated near the surface of the silver films, resulting in the increasing possibility of oxidation. Besides, the electrical resistivity of silver oxidation is 5.2 × 10^−5^Ω m [22] that is much larger than that of pure silver (1.6 × 10^−8^Ω m). So the silver oxidation may cause the increase of the electrical resistivity. The second one was the removal of the carbon that bridged the nanoparticles [13]. When *D* decreased, the accumulation of heat became faster and the curing depth became deeper, the carbon inside the films may be removed because of the increasing temperature. As a result, the contact between the silver particles got worse leading to the increasing electrical resistivity.

**Fig. 4:**
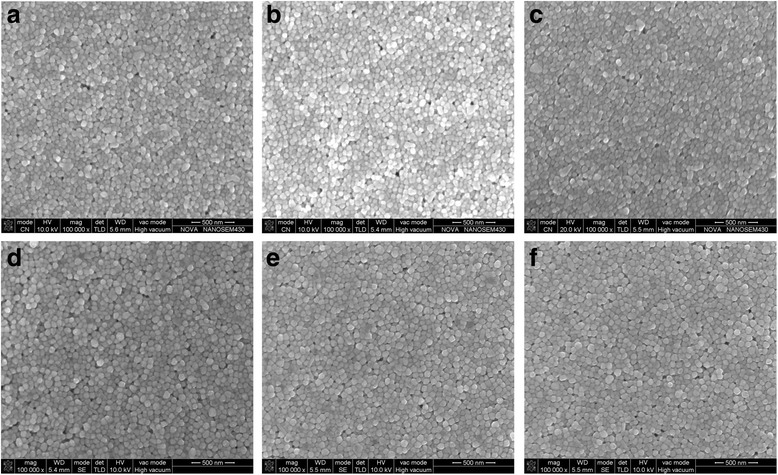
SEM images of silver films UV-cured at (**a**) *D* = 37 cm for 180 s; (**b**) *D* = 37 cm for 300 s; (**c**) *D* = 37 cm for 480 s; (**d**) *D* = 29 cm for 180 s; (**e**) *D* = 25 cm for 180 s; (**f**) *D* = 25 cm for 480 s

Figure 4 shows the SEM images of the silver films cured at different conditions. No obvious differences in the dispersion and size of the silver nanoparticles UV-cured at different conditions were observed. The nanoparticles with a uniform diameter were distributed uniformly on the surface and closely connected to each other, which indicated that the surface of the silver film was completely cured within a short time. It was curing depth and curing degree at different depth of the films that made the electrical resistivity of the silver films different.

**Fig. 5:**
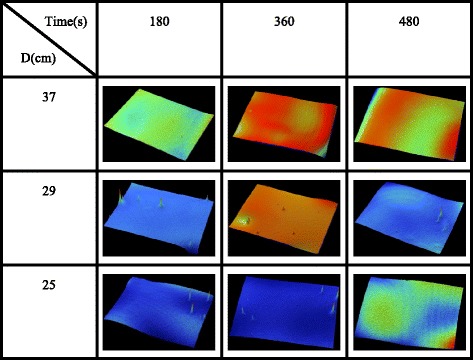
3D surface morphologies of the silver films at different UV curing conditions

Figure 5 shows the surface morphologies of the silver films UV-cured at different conditions. Several dispersed peaks appeared on the surface of the silver films when *D* was changed between 29 and 25 cm. However, there was no little peak when *D* = 37 cm. It meant that the curing depth increased as the decrease of *D*. When curing depth was too small to eliminate all organic solvents in inkjet-printed silver film, only the organic substances nearing to the surface were removed that had little effect on the surface morphology. But when curing depth was deep, the organic substances at deep depth had to break the shallow layer of the films to be removed leading to the appearance of the little peaks. So this phenomenon could also partially explain how *D* affected the electrical resistivity of the silver films.

According to above results, the electrical resistivity decreased as UV curing time increased up to 360 s, and then decreased slightly when UV curing time was larger than 360 s. Besides, the electrical resistivity also decreased when D decreased from 37 to 25 cm, but increased when *D* was smaller than 25 cm. So silver films were UV cured at different *D* ranging from 37 to 25 cm for different UV curing times in order to find out an optimum UV curing condition.

**Fig. 6:**
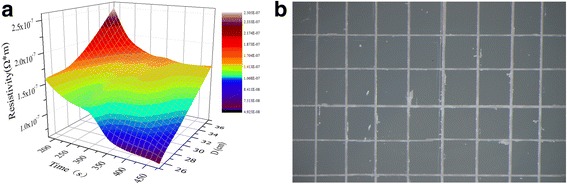
**a** 3D image for the electrical resistivity of the silver fims cured at different UV curing conditions. **b** Photo of UV-cured silver films on glass after tape test

Figure 6a shows the electrical resistivity of the silver films at different UV curing conditions. As shown in Fig. 6a, the electrical resistivity decreased with the increase of UV curing time at a specific *D* and also decreased with the decline of *D* at a specific UV curing time, which were consistent with Fig. 2a and Fig. 3a, respectively. We believed that the curing depth was affected by *D* while the curing degree was affected by both UV curing time and *D* according to the results from Fig. 5 and Fig. 6a. According to it, we prepared a silver gate electrode with low electrical resistivity (6.69 × 10^−8^Ω m) UV cured at *D* = 25 cm for 480 s. Besides, only a little part of the silver film was peeled off after tape test shown in Fig. 6b, exhibiting a good adhesion of 4B by the ASTM international standard.

**Fig. 7:**
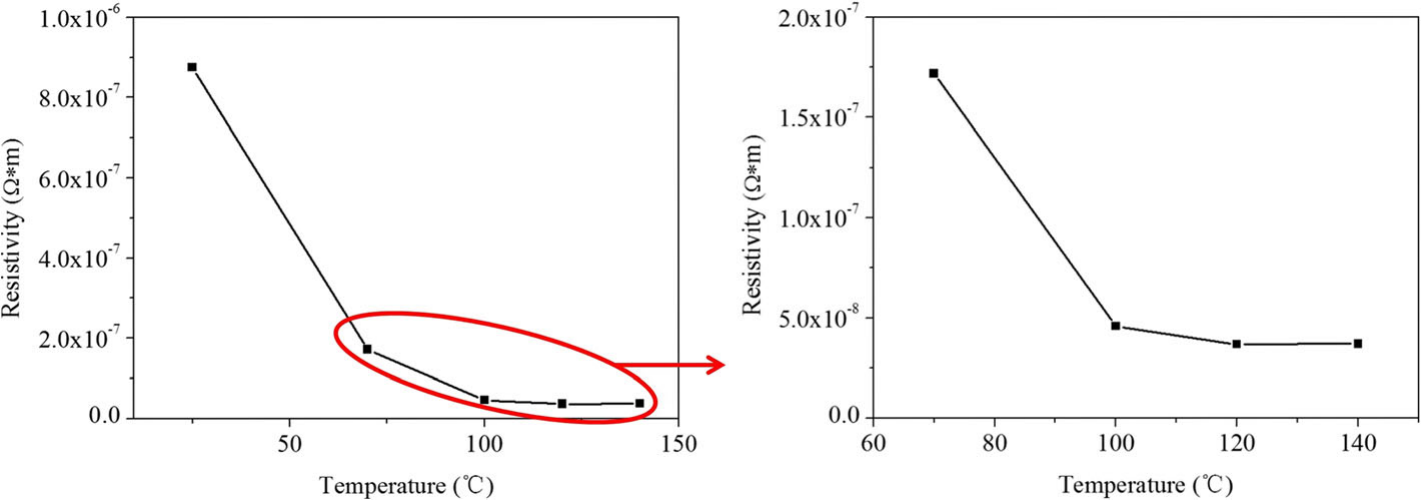
. Resistivity of silver films heat-treated at different temperatures for 30 min

**Fig. 8:**
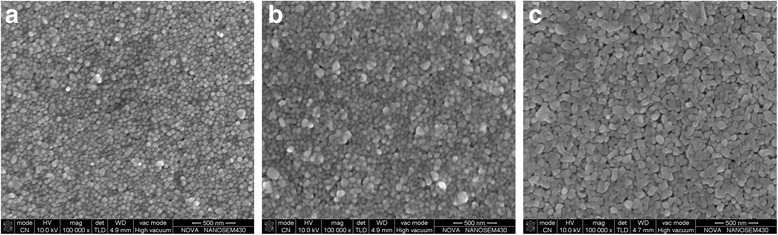
SEM images of the silver films heat-treated at different temperatures for 30 min: (**a**) 25 °C; (**b**) 100 °C; (**c**) 140 °C

**Fig. 9:**
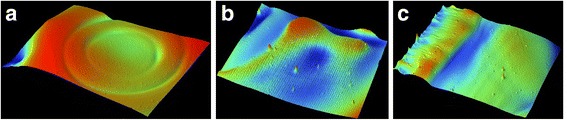
3D surface morphologies of the silver films heat-treated at (**a**) 25 °C, (**b**) 100 °C, and (**c**) 140 °C

In comparison to UV curing method, heat curing method was applied to treat the silver films at different temperatures. As shown in Fig. 7, the electrical resistivity decreased as the rise of temperature, but the electrical resistivity almost retained the same after the temperature is over 120 °C, with an electrical resistivity of 3.68 × 10^−8 Ω^ m. As shown in Fig. 8, the average size of nanoparticles gradually becomes larger with increasing temperature. A lot of nanoparticles started to merge into bigger particles when temperature reached 100 °C, and became coalesced together when temperature was 140 °C. Comparing Fig. 4 to Fig. 8, the nanoparticles of the heat-treated silver films were not as uniform as the UV-cured silver films. The electrical resistivity of the film UV-cured at *D* = 25 cm for 480 s was only about twice larger than that of the film heat-treated at 120 °C. We also could see that the UV-cured films were much smoother than heat-treated films by comparing Fig. 5 to Fig. 9. Moreover, the silver nanoparticles in the UV-cured films did not merged into bigger particles and there were few aggregations of the silver nanoparticles, which indicated that the temperature of UV curing was lower than that during heat curing. Besides, UV curing method was less time-consuming. So we believed that the fabrication of silver gate electrodes with low electrical resistivity at low temperature by UV curing method was feasible.

## Conclusions

In this work, we prepared inkjet-printed silver gate electrodes with an electrical resistivity of 6.69 × 10^−8^Ω m by UV radiation at *D* = 25 cm for 480 s. The effects of UV curing time and *D* on the electrical resistivity of silver nanoparticle films were investigated systematically. The electrical resistivity of silver films decreased as the UV curing time increased or the *D* decreased due to the efficient removal of organic substances. But when *D* was smaller than 25 cm, the electrical resistivity increased because of the possible oxidation of silver or the possible removal of the conductive carbon generated during UV curing. In comparison to the silver film cured by heat curing, the texture of UV-cured silver film is smoother than the heat-treated silver film; moreover, UV curing was less time-consuming. UV radiation provides a time-saving and efficient approach to fabricate the silver nanoparticle gate electrode with low electrical resistivity by UV curing method.
